# Accuracy of 3D-printed surgical guides compared with freehand technique for temporary anchorage device placement in the mandibular buccal shelf

**DOI:** 10.3389/fdmed.2026.1691232

**Published:** 2026-02-02

**Authors:** Yasmin Youssef, Bayan Alyammahi, Amar Hassan Khamis, Abdel Rahman Tawfik, Ahmed Ghoneima

**Affiliations:** 1 Department of Orthodontics and Pediatric Dentistry, Hamdan Bin Mohammed College of Dental Medicine (HBMCDM), Mohammed Bin Rashid University of Medicine and Health Sciences (MBRU), Dubai, United Arab Emirates; 2 Section of Orthodontics, School of Dentistry, University of California, Los Angeles (UCLA), Los Angeles, CA, United States

**Keywords:** 3D printing, mandibular buccal shelf, orthodontic anchorage, surgical guide, temporary anchorage devices (TADs)

## Abstract

**Objective:**

This *in vitro* study evaluated the accuracy of 3D-printed surgical guides for the placement of temporary anchorage devices (TADs) in the mandibular buccal shelf.

**Methods:**

Fifty CBCT scans from the Dubai Dental Hospital database were used to create 100 3D-printed mandibular models. A total of 100 TADs (BENEfit “R” screw, 11 mm length, 2 mm diameter) were inserted in the buccal shelf region between the first and second molars. Each mandible was duplicated to form two groups: (1) a guided group, where TADs were placed using a custom-designed surgical guide, and a (2) manual group, where placement was performed freehand.

**Results:**

The guided group showed significantly higher TAD placement accuracy than the manual group, with improved parallelism to roots, closer proximity to the buccal cortical bone, greater distance from the lingual cortical bone and root apices, more upright angulation, and increased safety from the inferior alveolar nerve (*p* < 0.05). Placement success was 98% vs. 70% for guided and manual groups, respectively (*p* < 0.001). Bone measurements showed greater width at the second molar and greater length at the first molar. Correlation analyses indicated that TAD angulation and proximity to cortical bone, root apices, and the inferior alveolar nerve were positively associated with local bone width, particularly at the second molar region (*p* < 0.05).

**Conclusion:**

The use of 3D-printed surgical guides significantly enhanced the accuracy, safety, and clinical reliability of TAD placement in the mandibular buccal shelf, offering a valuable adjunct to orthodontic practice.

## Introduction

Anchorage, defined as the resistance to unwanted tooth movement, is essential for the orthodontic management of both dental and skeletal malocclusions. Temporary anchorage devices (miniscrews) have gained increasing popularity in recent years, as they offer reliable anchorage control while minimizing the need for patient compliance. Several anatomical sites have been proposed for miniscrew insertion, including the palate, retromolar area, and the buccal alveolar processes of both the maxilla and mandible. Among these, the mandibular buccal shelf is considered a preferred site, particularly in the correction of Class III malocclusions, due to its thick cortical bone and safe distance from adjacent vital structures (1–3).

The placement of miniscrews requires high accuracy to prevent complications such as root damage, soft tissue irritation, or injury to the inferior alveolar nerve. Improper angulation or depth may compromise primary stability, reduce success rates, and lead to clinical failures. Careful preoperative assessment of bone quality, thickness, and proximity to adjacent anatomical structures is therefore essential. Cone-beam computed tomography (CBCT) provides a three-dimensional (3D) evaluation of bone morphology and anatomical relationships, offering superior diagnostic accuracy compared to traditional two-dimensional radiographs. CBCT allows clinicians to assess cortical bone thickness, interradicular space, and proximity to critical structures, thereby improving treatment planning and reducing the risk of iatrogenic complications (4, 5).

Customized surgical guides have recently been proposed as a method to accurately translate radiographic planning into clinical execution, thereby minimizing the risk of root or nerve injury, enhancing TAD placement precision, and enabling controlled angulation and optimal positioning (6–10). However, miniscrew failure can still occur due to anatomical factors, poor bone quality, or improper insertion technique, particularly in regions characterized by thin cortical bone or non-keratinized mucosa (11, 12). While 3D-printed surgical guides offer clear advantages in terms of precision and safety, evidence regarding their effectiveness in the mandibular buccal shelf remains limited. Given the clinical importance of this site, especially for providing skeletal anchorage in the management of Class III malocclusions, extensive investigation is necessary. The aim of the current study was to explore the role of a 3D-printed surgical guide in enhancing the accuracy, safety, and reliability of TAD placement in the mandibular buccal shelf.

## Materials and methods

Fifty CBCT scans were obtained from the Dubai Dental Hospital database according to predefined inclusion and exclusion criteria. Mandibles were included if they exhibited well-developed anatomical structures, clearly visible inferior alveolar nerve (IAN), and fully erupted first and second molars with complete root formation. Mandibles with unerupted first or second molars or with any pathological findings were excluded.

Segmented mandibles were generated using Relu software (Version 5.0.2, Relu BV, Leuven, Belgium) and each mandible was printed twice with a NextDent 5100 3D printer (NextDent 5100, NextDent B.V., Soesterberg, Netherlands). This resulted in 100 mandibular models. A total of 100 temporary anchorage devices (BENEfit “R” Screw, 11 mm length, 2 mm diameter, Germany) were placed in the buccal shelf region between the first and second mandibular molars. The sample size was calculated for a paired comparison (*α* = 0.05; power = 80%), using an effect size of 0.82 derived from Iodice et al. (10). Based on these assumptions, 50 mandibular models per group were deemed adequate.

**Figure 1:**
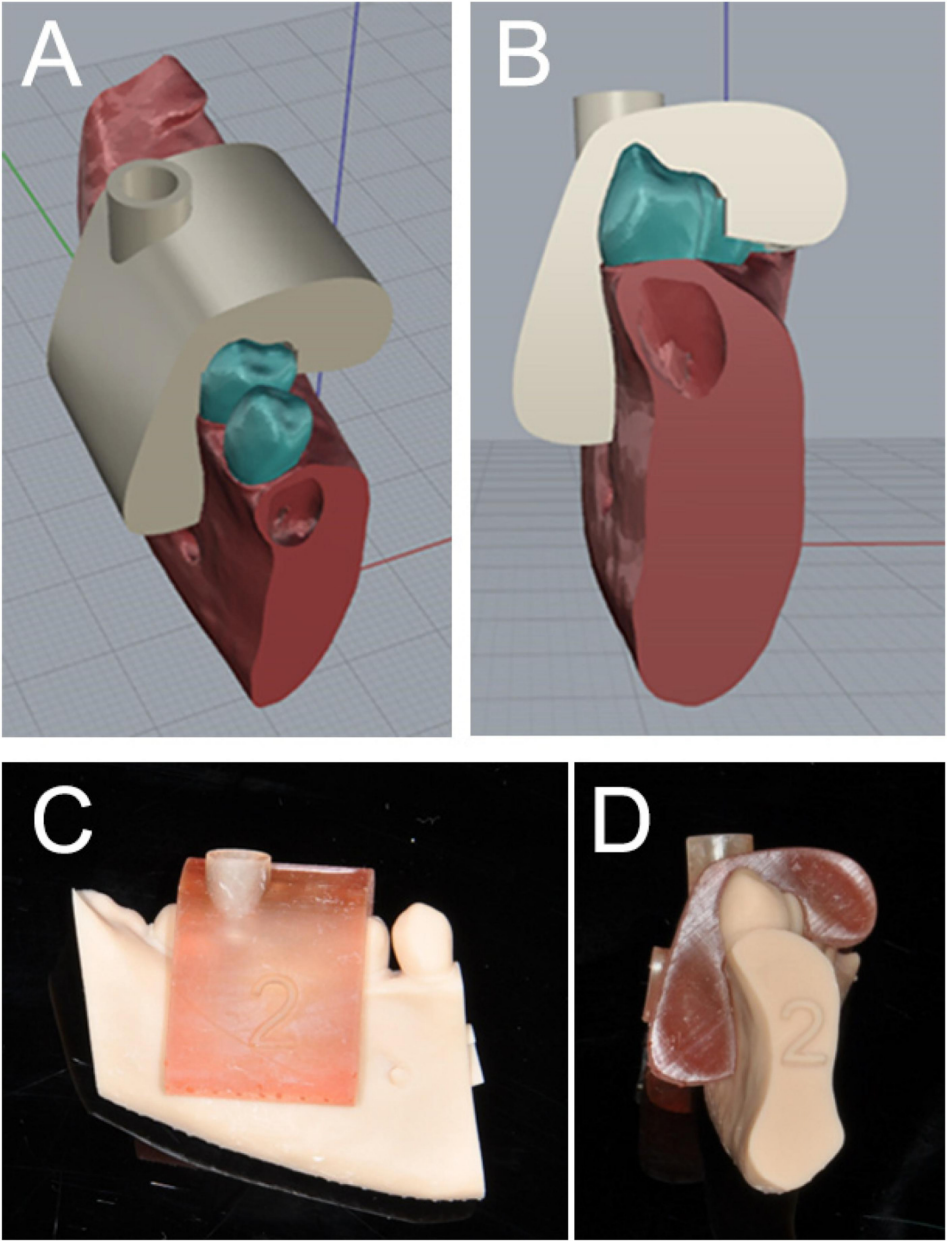
**(A,B)** surgical guide virtually designed with a guiding tube (5.5 mm-width and 7 mm-length). **(C,D)** The 3D printed surgical guide fitting on the mandible.

A custom surgical guide was designed using Rhino 3D software (version 5; McNeel North America, Seattle, WA, USA). The dimensions of the customised guide (5.5 mm width × 7 mm length) were determined based on the physical dimensions of the screwdriver used for temporary anchorage device (TAD) placement. The internal diameter and height of the guide sleeve were designed to accommodate the screwdriver shaft while providing adequate stability, controlling the insertion angulation, and facilitating smooth TAD insertion. A clearance of 0.5 mm greater than the screwdriver head diameter was incorporated to allow unobstructed passage of the screwdriver while minimising play during insertion. The guide was fabricated using NextDent SG surgical guide material with the same 3D printer (Figure 1). Post-processing included cleaning with 99% alcohol, air drying, and light curing for 10 min using a 3D Print Box curing unit to ensure optimal material properties and dimensional accuracy.

**Figure 2:**
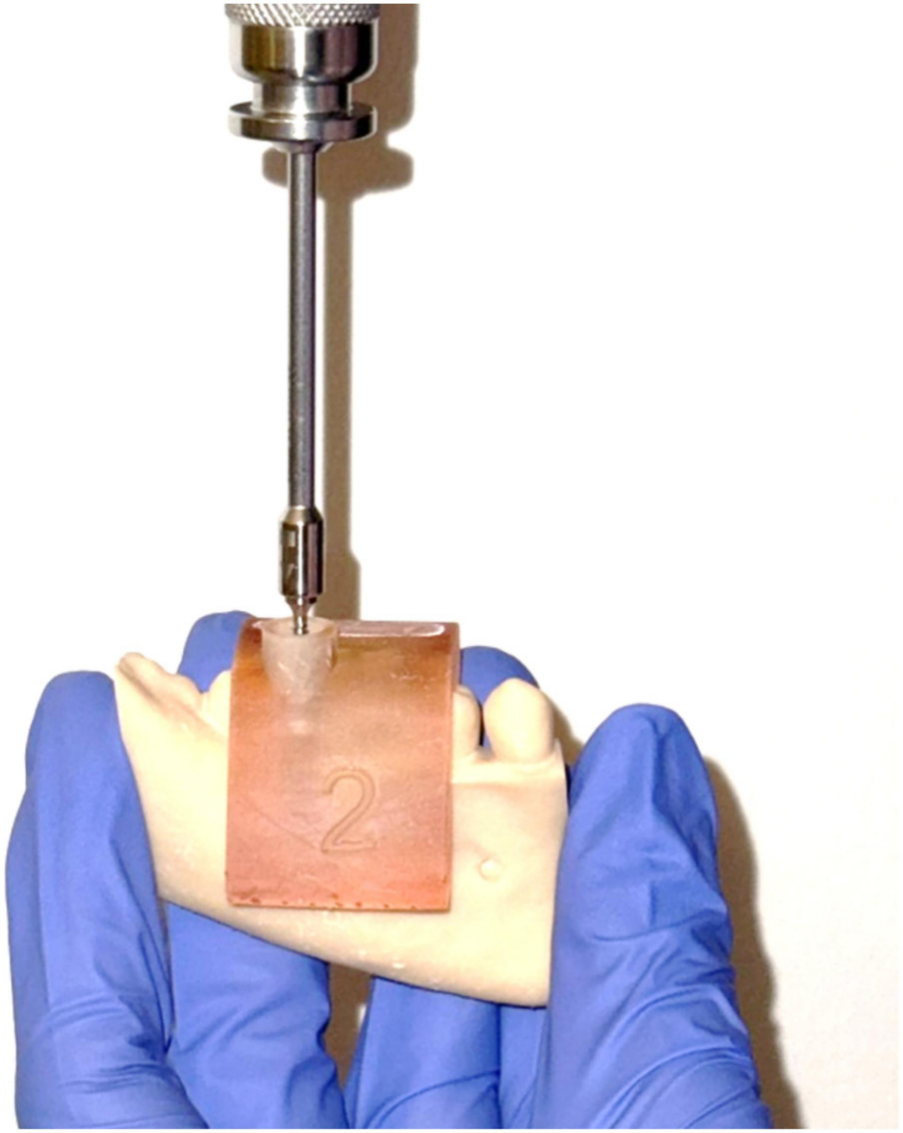
Insertion of TAD using the surgical guide.

Each mandible was used under two experimental conditions, either manual placement where TADs were inserted freehand using a 2 mm pilot hole drilled with a 1.4 × 33 mm drill, followed by manual insertion with a screwdriver aiming to align them as parallel as possible to the first and second molars, or guided placement where TADs were inserted with the aid of the 3D-printed surgical guide to control site and angulation. TAD angulation was individually customized for each guide to avoid contact with vital structures. Once half of the screw was inserted, the guide was removed, and insertion was completed manually. All insertions were performed by the same trained investigator (Y.Y.) to ensure consistency (Figure 2).

**Figure 3:**
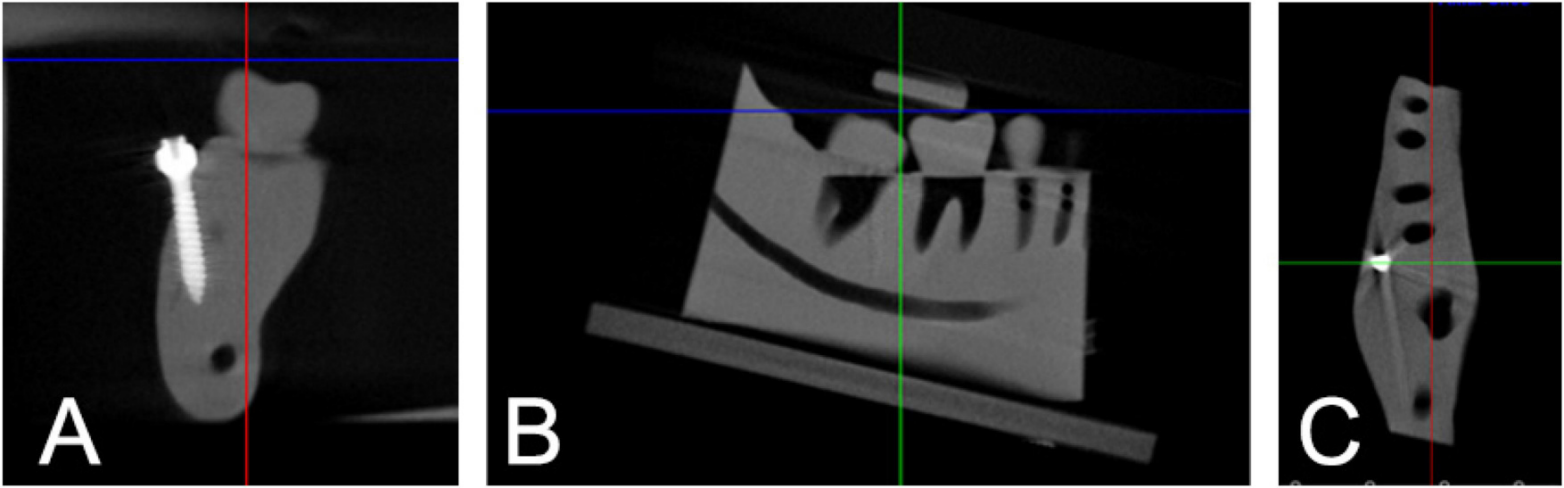
CBCT scans oriented three-dimensionally from **(A)** coronal, **(B)** sagittal, and **(C)** axial sections.

Following TAD placement, CBCT scans (Veraviewepocs 3D R100, Kyoto, Japan) were obtained for the right side of each model. Scans were converted to DICOM format and imported into Dolphin 3D software (version 11.95, Dolphin Imaging, Chatsworth, CA). All scans were standardized by orienting the distobuccal cusp of the second molar and the buccal cusp of the first premolar parallel to the axial plane, with the long axis of the alveolar ridge perpendicular to the axial plane (Figure 3).

**Figure 4:**
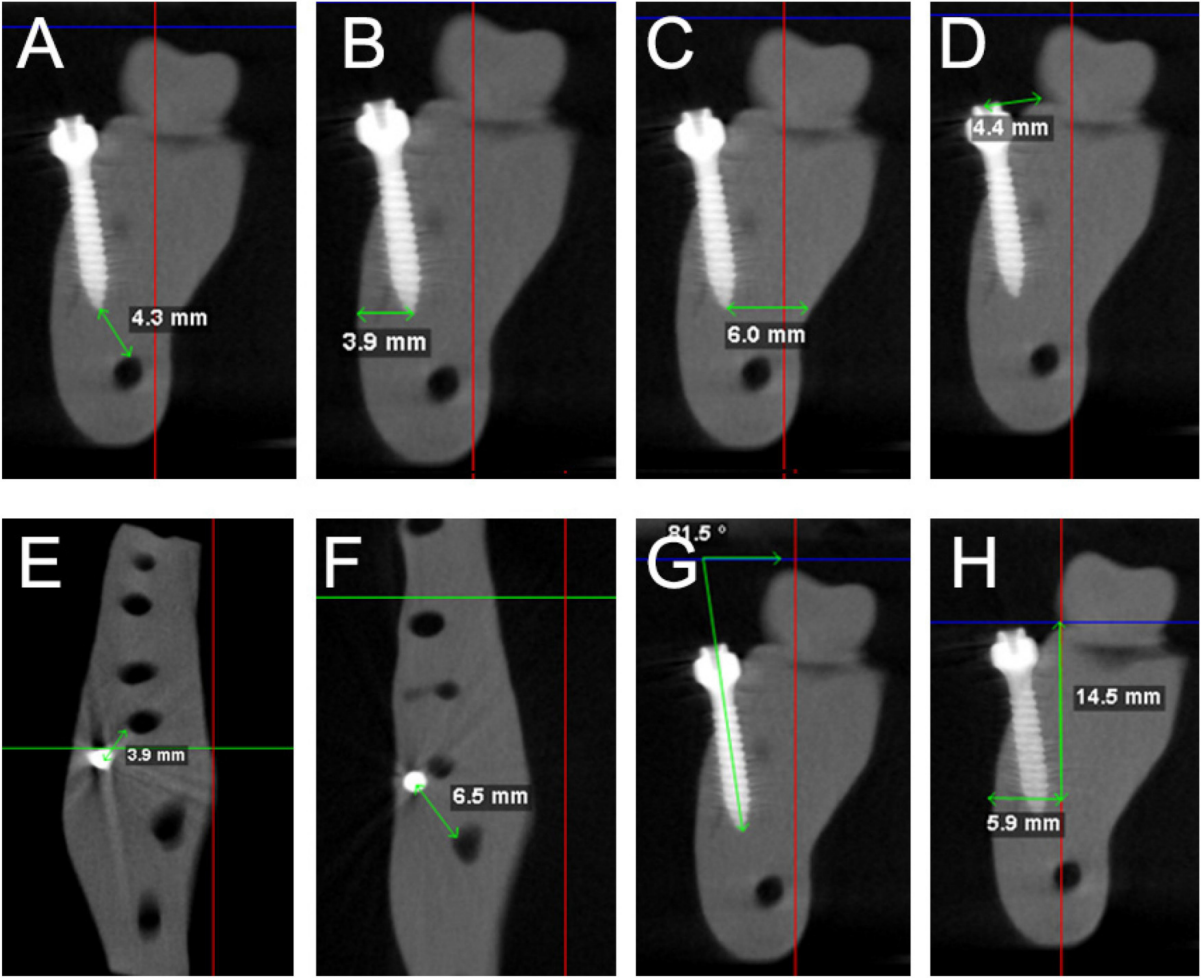
Measurements selected in this study; **(A)** distance between the tip of the TAD to the IAN, **(B)** between the tip of the TAD to the buccal cortical bone, **(C)** between the tip of the TAD to the lingual cortical bone, **(D)** the head of the TAD to the CEJ of the 2nd molar, **(E)** between the tip of the TAD and the distal root of the 1st molar, **(F)** between the tip of the TAD to the mesial root of the 2nd molar, **(G)** TAD angulation, and **(H)** bone length and width between the 1st and 2nd molars.

Linear and angular measurements were performed using Dolphin software. Six linear parameters were assessed (Table 1; Figure 4), along with bone length and width at the first and second molar regions.

**Table 1:** Parameters definitions.

Parameters	Definition
TAD-IAN	Distance from the tip of the TAD to IAN (coronal view)
TAD- buccal cortical bone	Distance from the tip of the TAD to the buccal cortical bone (coronal view)
TAD- lingual cortical bone	Distance from the tip of the TAD to the lingual cortical bone (coronal view)
TAD-CEJ of the 2nd molar	Distance from the head of the TAD to the cementoenamel junction of the second molar (coronal view)
TAD- distal root apex of the 1st molar	Distance from the tip of the TAD to the distal root of the first molar (axial view)
TAD- mesial root apex of the 2nd molar	Distance from the tip of the TAD to the mesial root of the second molar (axial view)
TAD angulation	An angular parameter recording the angle between the long axis of the screw and the alveolar ridge to assess the angle of insertion of each screw (coronal view)
Bone length and width	Assessment of bone length and width between the 1st and 2nd molars (coronal view)

Intra-rater reliability was tested by repeating all measurements twice on 10 randomly selected models from each group, with a one-week interval. All measurements were performed by the same examiner (Y.Y.). Internal consistency was assessed using paired t-tests. Data were analyzed using SPSS for Windows, version 29.0 (SPSS Inc., Chicago, IL). Continuous variables were reported as means and standard deviations. Normality was assessed using the Kolmogorov–Smirnov test. Depending on distribution, comparisons between groups were performed using independent t-tests or Mann–Whitney tests. For comparisons involving more than two means, ANOVA or Kruskal–Wallis tests were applied. Categorical variables were analyzed using Chi-square or Fisher's exact tests, as appropriate. Linear regression analysis was conducted to identify predictors of continuous dependent variables. A *p*-value < 0.05 was considered statistically significant.

## Results

The descriptive measurements of all assessed parameters are presented in Table 2. Overall, the use of a 3D-printed surgical guide demonstrated superior accuracy and safer positioning compared with manual placement. Guided TAD placement achieved higher angulation relative to the occlusal plane (76.90° ± 8.82 vs. 66.43° ± 11.05) with less variability compared to manual insertion. Guided placement positioned screws closer to the buccal cortical bone (4.11 ± 1.20 mm vs. 5.96 ± 1.54 mm) and farther from the lingual plate (8.13 ± 2.48 mm vs. 6.67 ± 2.33 mm). Greater clearance was also observed from adjacent roots, including the mesial root apex of the second molar (6.79 ± 1.87 mm vs. 5.77 ± 1.55 mm) and the distal root apex of the first molar (3.65 ± 1.67 mm vs. 2.17 ± 1.61 mm). The distance to the CEJ of the second molar was slightly lower in the guided group (5.61 ± 0.96 mm vs. 6.31 ± 1.17 mm), and clearance from the inferior alveolar nerve was improved (4.64 ± 1.89 mm vs. 3.32 ± 3.44 mm).

**Table 2:** Descriptive measurements of all the parameters selected in this study.

Parameters	Manual					Guide				
	Mean	SD	Min	Max	SE	Mean	SD	Min	Max	SE
TAD angulation	66.43	11.05	40.60	88.40	1.56	76.90	8.82	55.2	89.2	1.25
TAD-buccal cortical bone	5.96	1.54	3.2	9.4	0.22	4.11	1.20	1.7	7.5	0.17
TAD-lingual cortical bone	6.67	2.33	0.1	11.4	0.33	8.13	2.48	1.6	14	0.35
TAD-mesial root apex of the 2nd molar	5.77	1.55	0	9	0.22	6.79	1.87	1.9	10.2	0.26
TAD-distal root apex of the 1st molar	2.17	1.61	0	5.3	0.17	3.65	1.67	0	7.3	0.14
TAD-CEJ of the 2nd molar	6.31	1.17	4.3	9.8	0.49	5.61	0.96	3.3	7.5	0.27
TAD-IAN	3.32	3.44	−6.60	8.50	0.23	4.64	1.89	−2.6	9	0.24

Statistical comparisons between groups confirmed these differences to be significant across all parameters (Table 3). Guided placement resulted in greater angulation control (mean difference = −10.47°, *p* = 0.001), improved proximity to the buccal cortical plate (mean difference = 1.94 mm, *p* = 0.001), and increased clearance from the lingual cortical plate (mean difference = −1.37 mm, *p* = 0.005). Distances to the mesial root apex of the second molar (mean difference = −1.03 mm, *p* = 0.003), distal root apex of the first molar (mean difference = −1.48 mm, *p* = 0.001), and IAN (mean difference = −1.31 mm, *p* = 0.001) were all significantly improved with guided insertion. The reduction in distance to the CEJ of the second molar (mean difference = 0.70 mm) was also significant (*p* = 0.022). Additionally, the guided group achieved a 98% success rate compared with 70% in the manual group with statistically significant difference (*p* = 0.001) (Table 4).

**Table 3:** Difference between the manual and guided groups.

Parameters	Difference between manual and guide					95% confidence interval		*P*-value
	Mean	SD	Min	Max	SE	Upper	Lower	
TAD angulation	−10.47	9.61	−32.10	5.70	1.36	−7.74	−13.20	0.001^a^
TAD-buccal cortical bone	1.94	1.68	−0.80	6.20	0.24	2.42	1.47	0.001^a^
TAD-lingual cortical bone	−1.37	1.71	−4.70	3.30	0.24	−0.88	−1.85	0.005^a^
TAD-mesial root apex of the 2nd molar	−1.03	1.46	−4.90	1.50	0.21	−0.61	−1.44	0.003^a^
TAD-distal root apex of the 1st molar	−1.48	1.67	−5.60	2.70	0.24	−1.00	−1.95	0.001^a^
TAD-CEJ of 2nd molar	0.70	1.20	−2.20	3.30	0.17	1.04	0.36	0.022^a^
TAD-IAN	−1.31	3.64	−13.50	7.80	0.52	−0.28	−2.35	0.001^a^

a Significant at ≤0.05.

**Table 4:** Success rate of manual TAD placement vs. guide TAD placement.

Methods	Number of successful TADs	Percentage	Number of unsuccessful TADs	Percentage	*P*-value
Manual	35	70%	15	30%	0.001
Guide	49	98%	1	2%	0.001

a Significant at ≤0.05.

The descriptive analysis of bone dimensions showed that the mean bone length at the distal root of the first molar was 12.43 ± 2.88 mm, with a mean width of 3.94 ± 1.58 mm. At the mesial root of the second molar, the mean bone length was 11.29 ± 1.92 mm and the mean width was 5.53 ± 1.61 mm (Table 5). TAD angulation demonstrated significant positive correlations with bone width at the distal root of the first molar (*r* = 0.335, *p* = 0.018) and bone width at the mesial root of the second molar (*r* = 0.485, *p* = 0.001). The distance between the TAD and the lingual cortical plate was also significantly correlated with both bone width at the distal root of the first molar (*r* = 0.389, *p* = 0.005) and mesial root of the second molar (*r* = 0.549, *p* = 0.001). Similarly, the distance from the TAD to the mesial root apex of the second molar correlated positively with bone width at the distal root of the first molar (*r* = 0.302, *p* = 0.033). A significant positive correlation was also observed between TAD clearance from the distal root apex of the first molar and bone width at the mesial root of the second molar (*r* = 0.336, *p* = 0.017). TAD clearance from the inferior alveolar nerve showed a strong correlation with bone width at the mesial root of the second molar (*r* = 0.383, *p* = 0.006) (Table 6).

**Table 5:** Descriptive measurements of the bone width and length.

Parameters	Mean	SD	Minimum	Maximum
Length of bone at distal root of 1st molar	12.43	2.88	.0	17.30
Width of bone at distal root of 1st molar	3.94	1.58	.0	7.30
Length of bone at mesial root of 2nd molar	11.29	1.92	7.40	16.00
Width of bone at mesial root of 2nd molar	5.53	1.61	1.90	9.80

**Table 6:** Correlations between all parameters and bone width and length.

Parameters		Length of bone at distal root of 1st molar	Width of bone at distal root of 1st molar	Length of bone at mesial root of 2nd molar	Width of bone at mesial root of 2nd molar
TAD angulation	Pearson Correlation	.189	.335^a^	.081	.485^b^
	Sig. (2-tailed)	.190	.018	.574	0.001
TAD-buccal cortical bone	Pearson Correlation	.154	.230	.145	.085
	Sig. (2-tailed)	.285	.109	.315	.559
TAD and the lingual cortical bone	Pearson Correlation	.184	.389^b^	.169	.549^b^
	Sig. (2-tailed)	.200	.005	.239	0.001
TAD-mesial root apex of the 2nd molar	Pearson Correlation	.077	.302^a^	.158	.273
	Sig. (2-tailed)	.593	.033	.272	.055
TAD-distal root apex of the 1st molar	Pearson Correlation	.083	.179	−.069	.336^a^
	Sig. (2-tailed)	.565	.213	.636	.017
TAD-CEJ of 2nd molar	Pearson Correlation	−.193	.071	−.059	.075
	Sig. (2-tailed)	.180	.623	.686	.603
TAD-IAN	Pearson Correlation	.028	.080	.112	.383^b^
	Sig. (2-tailed)	.845	.579	.439	.006

a Correlation is significant at the 0.05 level (2-tailed).

b Correlation is significant at the 0.01 level (2-tailed).

## Discussion

The present study evaluated the accuracy of TAD placement in the mandibular buccal shelf using a 3D-printed surgical guide compared with conventional freehand insertion. Surgical guides enable precise and safe TAD placement in the mandibular buccal shelf by customizing the angulation and position according to each patient's anatomy. This approach minimizes the risk of root or nerve injury and enhances placement accuracy compared with manual insertion. To ensure consistency and minimize side to side variability, all pre- and post-placement CBCT analyses were confined to the right buccal shelf. The findings clearly demonstrate that guided placement enhances accuracy and minimizes the risk of iatrogenic complications. While the application of digital workflows and additive manufacturing in orthodontics is expanding, limited data exist on their role in skeletal anchorage. The aim of the present study was to assess the accuracy of 3D-printed surgical guides for the placement of TADs in the mandibular buccal shelf.

The improved angulation reported in the guided group of the study sample is consistent with the findings of Chang et al., who reported that miniscrews placed parallel and distal to the roots of the lower first molar had higher success rates in buccal shelf applications (13). In this study, guided TADs showed more parallel orientation to the adjacent roots, reducing variability compared to the freehand group. This angulation advantage ensured that the TAD apex was consistently positioned closer to the buccal cortical plate and further from the lingual plate. Similar benefits have been reported by Suzuki et al., who demonstrated that surgical guides reduce deviation and ensure accurate three-dimensional translation of the preoperative plan (14).

In terms of root safety, the guided technique consistently resulted in greater clearance from both the mesial root apex of the second molar and the distal root apex of the first molar. This is critically important as root contact remains one of the most frequent causes of TAD failure. These findings are in agreement with earlier reports indicating that the use of surgical guides minimized root injuries and improved placement accuracy (14, 15). Additionally, the current study findings demonstrated that guided placement reduced the risk of inferior alveolar nerve encroachment. Negative values recorded in the manual group indicated potential proximity to the IAN while guided placement consistently maintained safer margins. This observation emphasizes that digital planning with surgical guides not only improves accuracy but also enhances patient safety by protecting critical anatomical structures.

In this study, success was defined as the absence of post-placement contact between the TAD and vital structures, including the inferior alveolar nerve and adjacent tooth roots, and this criterion was applied consistently to both guided and manual placement groups. The clinical significance of these differences is indicated by the markedly higher success rate of TADs placed with guidance (98%) compared with manual placement (70%). Success in this context reflects not only the avoidance of contact with roots or neurovascular structures but also favorable angulation and cortical engagement, which are essential for primary stability.

The analysis of mandibular bone morphology revealed that bone width was greater at the second molar region compared with the first molar, which aligns with previous reports indicating that the buccal cortical bone thickness increases distally, reaching a maximum at the second molar region (16). The correlation analysis further demonstrated that greater bone width, particularly at the mesial root of the second molar, was associated with improved angulation, safer distances from roots, and increased clearance from the lingual cortical plate. These findings suggest that anatomical variability, especially buccolingual bone width, is a key determinant of TAD stability and accuracy. Guided placements seem to adapt more effectively to this variability by translating radiographic planning into clinically reproducible outcomes.

Findings from the present study support the clinical advantage of surgical guides in orthodontics. Freehand placement in the buccal shelf may be subject to variability due to anatomical restrictions, operator experience, and intraoral accessibility. In contrast, a digital workflow that incorporates CBCT analysis and 3D-printed guides allows for optimized site selection, controlled angulation, and reduced risk of complications. This is especially relevant for Class III cases, where mandibular buccal shelf anchorage is increasingly used as an alternative to extraoral anchorage or surgical intervention (17, 18).

The strength of this study relies on its use of standardized models derived from actual patient CBCT scans, which allowed for consistent evaluation of guided vs. manual placement under controlled conditions. The detailed linear and angular measurements provided a comprehensive analysis of TAD positioning relative to critical structures. However, multiple limitations must be acknowledged. The study was conducted using acrylic 3D-printed mandibular models, which lack the density and biomechanical properties of human bone. Although the models provided reproducibility, the absence of bone elasticity and soft tissue structures limited the simulation of clinical challenges such as insertion torque, tactile feedback, and soft tissue interference. Additionally, the lack of intraoral constraints (cheek, tongue, and access limitations) may have made the placement process less technically difficult than *in vivo* conditions. The customised surgical guide evaluated in this study has not been previously clinically validated. Therefore, the findings should be interpreted with caution, and further experimental and clinical validation studies are required before routine clinical implementation.

## Data Availability

The original contributions presented in the study are included in the article/Supplementary Material, further inquiries can be directed to the corresponding author.
